# Mapping the differential impact of spontaneous and conversational laughter on brain and mind: an fMRI study in autism

**DOI:** 10.1093/cercor/bhae199

**Published:** 2024-05-16

**Authors:** Ceci Qing Cai, Nadine Lavan, Sinead H Y Chen, Claire Z X Wang, Ozan Cem Ozturk, Roni Man Ying Chiu, Sam J Gilbert, Sarah J White, Sophie K Scott

**Affiliations:** Institute of Cognitive Neuroscience, University College London, London WC1N 3AZ, United Kingdom; Department of Biological and Experimental Psychology, School of Biological and Behavioural Sciences, Queen Mary University of London, London E1 4NS, United Kingdom; Institute of Cognitive Neuroscience, University College London, London WC1N 3AZ, United Kingdom; Institute of Cognitive Neuroscience, University College London, London WC1N 3AZ, United Kingdom; Institute of Cognitive Neuroscience, University College London, London WC1N 3AZ, United Kingdom; Department of Social and Behavioural Sciences, City University of Hong Kong, Tat Chee Avenue, Kowloon, Hong Kong SAR; Institute of Cognitive Neuroscience, University College London, London WC1N 3AZ, United Kingdom; Institute of Cognitive Neuroscience, University College London, London WC1N 3AZ, United Kingdom; Institute of Cognitive Neuroscience, University College London, London WC1N 3AZ, United Kingdom

**Keywords:** autism, fMRI, laughter, medial prefrontal cortex, social communication

## Abstract

Spontaneous and conversational laughter are important socio-emotional communicative signals. Neuroimaging findings suggest that non-autistic people engage in mentalizing to understand the meaning behind conversational laughter. Autistic people may thus face specific challenges in processing conversational laughter, due to their mentalizing difficulties. Using fMRI, we explored neural differences during implicit processing of these two types of laughter. Autistic and non-autistic adults passively listened to funny words, followed by spontaneous laughter, conversational laughter, or noise-vocoded vocalizations. Behaviourally, words plus spontaneous laughter were rated as funnier than words plus conversational laughter, and the groups did not differ. However, neuroimaging results showed that non-autistic adults exhibited greater medial prefrontal cortex activation while listening to words plus conversational laughter, than words plus genuine laughter, while autistic adults showed no difference in medial prefrontal cortex activity between these two laughter types. Our findings suggest a crucial role for the medial prefrontal cortex in understanding socio-emotionally ambiguous laughter via mentalizing. Our study also highlights the possibility that autistic people may face challenges in understanding the essence of the laughter we frequently encounter in everyday life, especially in processing conversational laughter that carries complex meaning and social ambiguity, potentially leading to social vulnerability. Therefore, we advocate for clearer communication with autistic people.

## Introduction

Autistic people often encounter difficulties in non-verbal social communication (Mundy et al. 1986). While most research in this area centers on visual cues, such as eye gaze, gesture and facial expressions, revealing different patterns between autistic and non-autistic people (Senju et al. 2009; Trevisan et al. 2018), auditory cues have largely been neglected. It is equally important to understand how autistic people perceive and experience non-verbal auditory cues differently, particularly in the context of positive emotional expressions. Indeed, the literature to date has mostly focussed on negative emotional expressions, such as anger, sadness, fear, disgust, with relatively limited attention paid to the expression of positive emotions (see the reviews by Uljarevic and Hamilton 2013 and Leung et al. 2022). A shift toward exploring the full range of emotional expressions would enhance our understanding of the social strengths and weaknesses of autistic people, promoting a more balanced perspective. Furthermore, by exploring the diverse social communication experiences of autistic people, we can gain insights into the underlying mechanisms of social communication and interaction.

Laughter, as a universal positive emotional expression, plays a significant role in social bonding during human interactions (Sauter et al. 2010; Bryant and Bainbridge 2022). Although laughter is often viewed as a spontaneous and uncontrolled emotional vocalization triggered by tickling and humor (Provine 2004; Gervais and Wilson 2005), it predominantly occurs in conversation as a voluntary communicative signal (Provine 1993; Vettin and Todt 2004): people frequently laugh after verbal utterances to signal affiliation and agreement with others, mediating the meaning of utterances, and regulating the flow of conversation (Vettin and Todt 2004). Here, we define spontaneous laughter as uncontrolled and involuntary, and conversational laughter as controlled and voluntary (Provine 2004; Gervais and Wilson 2005; McGettigan et al. 2015). Although the production of laughter varies in the degree of volitional control and emotional content, much of the laughter that occurs naturally is likely to be a mix of both types (Scott et al. 2022). Spontaneous and conversational laughter are therefore both salient social signals that can play very different roles in communicating socio-emotional meaning (Neves et al. 2017), recruit different production systems (Wild et al. 2003; Gerbella et al. 2020), and are perceived to differ in authenticity, associated with acoustic differences (Lavan et al. 2016). Laughter not only promotes group cohesion but also fosters rapport in human interactions (Dunbar et al. 2012; Manninen et al. 2017) and intimacy (Gray et al. 2015). Understanding the meaning of laughter in various social contexts is therefore essential for individuals to establish and maintain social bonds and relationships (Scott et al. 2014).

Human laughter is a highly contagious behavior (Provine 1992). The perception of laughter engages oro-facial mirror networks, including premotor cortex, the pre-supplementary motor area (SMA) and right inferior frontal gyrus (Warren et al. 2006; O’Nions et al. 2017), consistent with findings that much of human adult laughter is associated with behavioral contagion (Provine 1992)—we are primed to laugh when we hear laughter, and we are 30 times more likely to laugh when with others than when alone (Provine and Fischer 1989). The contagious-laughter effect is strongly mediated by social contexts, such as the audience size and the intimacy/familiarity of the relationship (Provine 1992; Scott et al. 2022). However, autistic people tend to express and experience laughter differently to their peers (Reddy et al. 2002; Hudenko et al. 2009; Wu et al. 2015; Helt and Fein 2016; Helt et al. 2020). For instance, autistic children are less likely to join in others’ laughter and are more prone to laughing by themselves; they seldom try to elicit laughter from others and are less inclined to laugh at funny faces or socially inappropriate act (Reddy et al. 2002). They perceive cartoons with a laugh track to be less enjoyable than one without, and laugh less when watching these cartoons than non-autistic children (Helt and Fein 2016). Laughter might be less socially contagious for autistic people.

The perception of laughter involves the engagement of high-level cognitive networks, such as the metalizing network (Szameitat et al. 2010; McGettigan et al. 2015; Lavan et al. 2017; Sumiya et al. 2017). However, the processing of laughter differs with respect to its authenticity (McGettigan et al. 2015; Lavan et al. 2017). When considering the processing of different *types* of laughter, passive listening to spontaneous laughter induces greater activity in auditory cortex (superior temporal gyri, STG) than conversational laughter, likely reflecting the processing of emotional authenticity between these types of laughter. Interestingly, conversational laughter specifically engages mentalizing networks, showing greater activation in the medial prefrontal cortex (mPFC) and anterior cingulate cortex (ACC) compared to spontaneous laughter (McGettigan et al. 2015), and the degree of mPFC activation correlates with the perceived authenticity of laughter (Lavan et al. 2017). This suggests that laughter processing also involves representing the intentions behind the laughter, especially with socially ambiguous conversational laughter. As autistic people experience difficulties in mentalizing, supported by neuroimaging evidence of atypical activation of mPFC (Frith and Frith 2006; Gilbert et al. 2009; White et al. 2014), they may specifically struggle to comprehend the meaning of conversational laughter, processing it more like spontaneous laughter, which may subsequently impact their use of laughter in social interactions. Indeed, autistic children rarely produce unvoiced laughter during social play, which is more closely associated with spontaneous than conversational laughter (Hudenko et al. 2009).

Experiencing and understanding laughter in real life may be different for autistic people due to its contagious nature and the richness in social meaning; however, studies focusing on the processing of laughter in autistic adults are rare. We have previously found laughter is implicitly processed similarly by both autistic and non-autistic people: not only by its presence but also by its type. By adding either spontaneous or conversational laughter to spoken jokes, laughter increases the funniness of “dad jokes”; additionally, spontaneous laughter amplifies the funniness of jokes more than conversational laughter, likely due to its greater authenticity (Cai et al. 2019). We further found that although both autistic and non-autistic adults could explicitly differentiate between these two types of laughter by rating the affective properties of laughter itself, autistic adults rated conversational laughter as more authentic and emotionally arousing than non-autistic adults, perceiving it to be more similar to spontaneous laughter (Cai et al. in press). This discrepancy in the processing of laughter, evident between implicit and explicit processing, suggests that autistic people do not universally experience difficulties with all forms of non-verbal cues; in our case, all types of laughter. Intriguingly, however, autistic adults may process conversational laughter to be more like spontaneous laughter, possibly as a result of mentalizing difficulties in interpreting the socio-emotional meanings embedded in conversational laughter.

Despite typical behavioral responses to laughter, an fMRI study of laughter in autistic adults found reduced mPFC activation to written jokes followed by laughter; while laughter increased the pleasantness of jokes for all participants, this effect was smaller in autistic adults (Sumiya et al. 2020). However, this study did not report what type of laughter was used. It is therefore unclear whether autistic adults would show reduced mentalizing-related activation during the processing of all types of laughter due to their atypical non-verbal communication, or whether autistic adults might only show atypical neural response to conversational laughter that contains a degree of social ambiguity, whilst perceiving spontaneous laughter similarly to non-autistic people. Given laughter’s salient role in social communication and bonding, understanding the neural similarities and differences in processing different *types* of laughter in autism is essential. This can reveal how autistic people process laughter as a socio-emotional signal in everyday contexts and help identify potential areas of social vulnerability during communication. To address this gap in the literature, we aim to further explore the neural systems recruited in the implicit processing of all types of laughter. Additionally, we aim to explore whether the profile of neural activity in implicit processing of different *types* of laughter is in line with previous findings of explicit processing of laughter, with a particular interest in the involvement of the mPFC in the processing conversational laughter. Using fMRI, both autistic and non-autistic adults passively listened to funny words followed by either spontaneous laughter, conversational laughter, or noise-vocoded (NV) human vocalization. Post-scan, participants listened to the word plus laughter pairs again and rated the funniness of each word. We hypothesized that there would be differences in the neural correlates of implicit processing of these different types of laughter between autistic and non-autistic adults, specifically in the mPFC when listening to conversational laughter, and in the sensorimotor network to both types of laughter, despite typical behavioral responses to laughter.

## Materials and methods

### Participants

Twenty-five autistic adults and 23 non-autistic adults participated in this study, they were right-handed and had no speech, hearing or neurological difficulties. The groups were comparable for sex (*χ*^2^(1) = 0.060, *P* = 0.807), age (*t*(46) = −.134, *P* = 0.894), and verbal (*t*(46) = 0.720, *P* = 0.475), performance (*t*(46) = −.875, *P* =. 386), and full-scale IQ (*t*(46) = 0.031, *P* = 0.975), as measured by four subtests of the Wechsler Adult Intelligence Scale (WAIS-IV UK; Wechsler 2008: Matrix Reasoning, Block Design, Similarities, Vocabulary). The groups differed on Autism Spectrum Quotient (AQ; Baron-Cohen et al. 2001), *t*(46) = 11.879, *P* < 0.001. Although two autistic males were unable to complete the scan due to discomfort and health concerns, the scan groups remained comparable for sex (*χ*^2^(1) = 0.000, *P* = 1.000), age (*t*(44) = − 0.258, *P* = 0.798), and verbal (*t*(44) = 0.392, *P* = 0.697), performance (*t*(44) = −1.123, *P* =. 268), and full-scale IQ (*t*(44) = −.303, *P* = 0.763), and differed on AQ, *t*(44) = −12.325, *P* < 0.001. See Table 1.

**Table 1:** Participant demographic information.

	**NA**	**Autism**	**Autism for scan**
*N* (male: female)	23 (13:10)	25 (15:10)	23 (13:10)
Age (years)	28.348 (9.203)	28.720 (9.969)	29.087 (10.238)
Verbal IQ	121.435 (15.048)	117.800 (19.416)	119.435 (19.315)
Performance IQ	109.478 (15.985)	113.560 (16.289)	114.783 (16.059)
Full Scale IQ	119.000 (15.895)	118.840 (19.429)	120.565 (19.023)
AQ	13.652 (5.928)	35.560 (7.292)	37.348 (7.062)

*Note.* Values are given as mean (standard deviation). NA = non-autistic; AQ = autism-spectrum quotient.

The autistic participants had received an official diagnosis from a qualified clinician. Due to testing restrictions during the COVID-19 pandemic, we were unable to administer ADOS (Hus and Lord 2014) to confirm their diagnosis. Nonetheless, the AQ was used in the pre-screening assessment, and non-autistic participants with an AQ score below the cut-off of 32 were included in the study. Non-autistic participants were recruited from local participant databases. Autistic participants were recruited through university disability services and autism databases throughout the c. Informed written consent was obtained prior to testing, and the project received approval from the university research ethics committee.

### Experimental design and procedure

#### Word stimuli

A subset of 300 words was selected from the original pool (Engelthaler and Hills 2018). The words were chosen to avoid floor and ceiling effects, as per the results of the baseline ratings task (funniness: mean = 3.309, *SD* = 0.586; intelligibility: mean = 92.959%, *SD* = 10.619%), and recorded by a professional male comedian, who read the words in a comedic manner (duration: mean = 0.736 s, *SD* = 0.225 s; root-mean-square: mean = 0.031, *SD* = 0.000; pitch: mean = 189.677 Hz, *SD* = 82.154 Hz). Full details of word selection are given below.

To avoid the floor effect, we initially selected 719 words with a humor rating higher than 2.8 from a 5-point Likert scale (1—humorless; 5—humorous) from original pool (Engelthaler and Hills 2018). Subsequently, a list of 621 words was screened by four native English speakers to ensure appropriateness. The raw audio file was downsampled at a rate of 44,100 Hz to mono.wav files with 32-bit resolution, and each word was trimmed and edited into a 1-s sound file (.wav) using version 2.3.3 of Audacity (R) recording and editing software. The files were then normalized for root-mean-square (RMS) amplitude using PRAAT (Boersma, 2021). We further conducted an online task to establish baseline ratings for the funniness of words. In this task, 621 words were assigned to three lists, each containing 207 words. The three lists were matched on the humor ratings from the original pool (List 1: *M* = 3.19, *SD* = 1.23; List 2: *M* = 3.20, *SD* = 1.23; List 3: *M* = 3.18, *SD* = 1.23). Fifty-eight native English speakers were randomly assigned to one of the three lists (list 1 *n* = 18; list 2 *n* = 19; list 3 *n* = 21). Participants were instructed to listen to the recordings of a comedian, called “Ben” (not his real name), performing some funny words and were asked to rate the funniness of each word on a 7-point rating scale (“How funny was the word the way that Ben said it?” 1—not funny at all, 4—neutral, 7—extremely funny). Additionally, participants indicated whether they understand the meaning of each word. A practice trial was given before the actual task. The task was built and presented using Gorilla Experiment Builder (Anwyl-Irvine et al. 2020).

#### Sound stimuli

This study used 150 sound stimuli: 50 spontaneous laughter stimuli, 50 conversational laughter stimuli, and 50 NV human vocalizations. The spontaneous and conversational laughter stimuli were recorded using a method previously validated in behavioral and neuroimaging experiments (McGettigan et al. 2015; O’Nions et al. 2017) and were selected from a previous study, as detailed in (Cai et al. 2019). We created NV stimuli by applying one-channel noise-vocoding to various human emotional vocalizations, including expressions of anger (eight clips), pleasure (six clips), disgust (six clips), surprise (three clips), achievement (four clips), contentment (eight clips), fear (five clips), relief (five clips), sad (five clips). The resulting NV stimuli lack emotional meaning and are not recognized by normal-hearing listeners. We normalized all stimuli for RMS amplitude using PRAAT (Boersma, 2021), and extracted the acoustic parameters. One-way ANOVA indicated that spontaneous laughter, conversational laughter and NV sound stimuli were comparable in duration, RMS, and intensity (Table 2). For the detailed comparation of acoustic properties between spontaneous and conversational laughter, see Supplementary Table S1.

**Table 2:** Acoustic properties of sound stimuli set.

Acoustic measure	Conditions	Mean	SD	*F*		*P*
Total duration (s)	Spont	2.376	.406	1.297		.276
	Conver	2.269	.361			
	NV	2.307	.216			
Root-mean-square	Spont	.317	.000	.908		.406
	Conver	.317	.000			
	NV	.317	.000			
Intensity (dB)	Spont	64.000	.000	.945		.391
	Conver	64.000	.000			
	NV	64.000	.000			

*Note. df* = (2, 147). *P*-values are given in two tailed. Spont = spontaneous laughter; Conver = conversational laughter; NV = noised-vocoded human vocalization.

#### fMRI experimental design

**Fig. 1:**
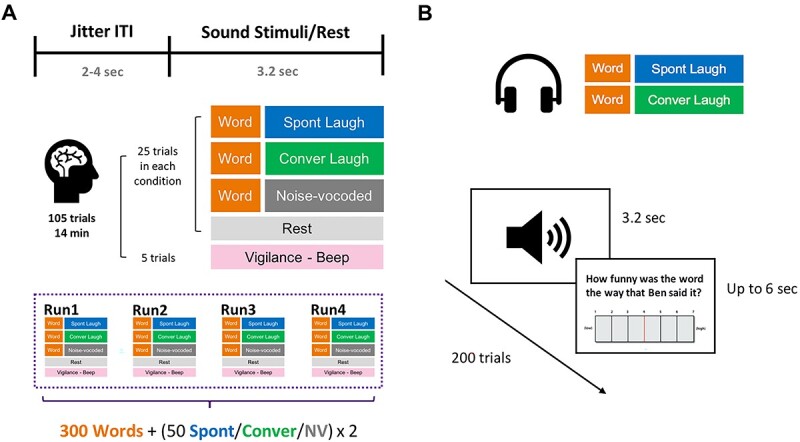
Experimental design of (A) scan session: implicit laughter processing and (B) post-scan behavioral session: Implicit laughter rating task. *Note.* Spont Laugh = spontaneous laughter; Conver Laugh = conversational laughter; Noised-vocoded = noised-vocoded human vocalization.

An event-related paradigm was utilized, with each trial consisting of a jittered inter-trial interval (ITI) ranging from 2 to 4 s. In the sound stimuli conditions, a funny word was presented, followed by a sound stimulus from one of three conditions (Spont Laugh, Conver Laugh, NV) with a fixed duration inter-stimulus interval (ISI) of 0.09 s. The rest condition included a 2-s period of silence following the ITI. Vigilance trials involved a 0.5-second beep sound, requiring participants to press a button within 3 s. Each functional run, approximately 14 min long, comprised 25 trials per condition and five vigilance trials to assess attentiveness. The entire experiment consisted of four functional runs with a 1-min rest period between runs, each comprising 105 trials, during which participants passively listened to 300 words paired with sound stimuli. The sound stimuli were used twice each. Trial conditions were pseudorandomized to prevent more than two consecutive trials of the same condition involving words plus sound stimuli. Furthermore, neither rest nor vigilance conditions were the first trial of each run, nor were they presented consecutively. The pairs of words plus sound stimuli were pseudorandomized and counterbalanced across both runs and participants (see Fig. 1A).

#### Behavioral experimental design

Participants listened to the 200 word plus laughter pairs again and this time also rated the funniness of each word. Due to time constraints imposed by COVID testing restrictions, the 100 word plus NV pairs were excluded. The pairs were the same as in the previous scan conditions, but the order of the pairs was shuffled. Participants rated each word on a 7-point rating scale (“How funny was the word the way that Ben said it?,” 1—not funny at all, 4—neutral, 7—extremely funny). For each trial, participants had up to 6 s to give a rating. There was a short practice session before the real task to let participants become familiar with the structure of the task. The post-scan behavioral task lasted approximately 25 min (see Fig. 1B).

#### Procedure

Participants were informed that the fMRI study was about humor processing. Notably, any mention of laughter was intentionally avoided during the recruitment and prior to testing. Before the scan, participants were instructed to listen to humorous words spoken by a comedian, and people’s reactions. They were told to press a button on a button-box whenever they heard a “beep” sound. A practice sequence at the beginning ensured the volume was adequate and that participants could clearly hear the stimuli by recalling the words they heard. The testing lasted approximately 2 h, split between a 1-h brain scan and a 1-h behavioral test session, which encompassed the post-scan behavioral task, IQ test, and questionnaires. Both fMRI and behavioral experiments were presented using MATLAB R2018B (MathWorks Inc 2018) with the psychophysics toolbox (Brainard 1997).

### Neuroimaging, pre-processing, and analysis

#### Acquisition

We employed continuous event-related fMRI, acquiring blood-oxygen-level-dependent (BOLD) images using a Siemens Avanto 1.5-Tesla MRI scanner with a 32-channel head coil. The study involved four runs of 260 echo-planer whole-brain volumes (TR = 3 s; TE = 50 ms; TA = 86 ms; Slice tilt = 25° ± 5°; flip angle = 90°; 3 mm × 3 mm × 3 mm in-plane resolution). Auditory stimuli were delivered via an MR-compatible insert earphone connected to a Sony STR-DH510 digital AV control center. After two functional runs, we obtained high-resolution anatomical images using a T1-weighted magnetization prepared-rapid acquisition gradient echo sequence (176 sagittal slices, TR = 2730 ms; TE = 3.57 ms; flip angle = 7°, acquisition matrix = 224 × 256 × 176, slice thickness = 1 mm, 1 mm × 1 mm × 1 mm).

#### Software

Pre-processing and statistical analysis were conducted in SPM12 (Penny et al. 2011), implemented in MATLAB R2018B (MathWorks Inc 2018).

#### Pre-processing

The first three volumes of each EPI sequence were discarded. The remaining volumes underwent spatial alignment along the AC-PC axis for each participant, followed by slice time correction using the last slice as a reference. The corrected images were then spatially realigned and registered to the mean. The structural image was co-registered with the mean of the corrected images, aligning structural scans with SPM12 (Penny et al. 2011) tissue probability maps during segmentation. The forward deformations image from the segmentation was used to normalize the functional images to standard MNI space. Finally, the normalized functional images were resampled into 2 × 2 × 2 mm voxels and spatially smoothed using an isotropic 8 mm full width at half-maximum Gaussian kernel.

#### Analysis

fMRI data were analyzed in an event-related manner. Variance in each time series was decomposed in a voxelwise general linear model with the following regressors: onsets and durations of (1) words plus spontaneous laughter (Spont Laugh), (2) words plus conversational laughter (Conver Laugh), (3) words plus NV stimuli (NV), and (4) vigilance trials. These regressors, along with six additional regressors representing realignment parameters calculated by SPM12 (Penny et al. 2011), constituted the full model for each session. The data were high-pass filtered at 128 s.

Individual design matrices were contrasted per participant [All Laughs (Spont Laugh & Conver Laugh) > NV, Spont Laugh > NV, Conver Laugh > NV], modeling the three experimental conditions across four runs and including movement parameters as nuisance variables. These contrasts were entered into a second level, two-sample t-test for the group analysis. Whole-brain analysis results were corrected for multiple comparisons using a cluster-extent-based thresholding approach (Poline et al. 1997): a voxel-wise threshold of *P* < 0.001 combined with a cluster extent threshold determined by SPM12 (Penny et al. 2011) (*P* < 0.05 family-wise-error cluster-corrected threshold). All reported clusters exceeded this cluster-corrected threshold. Reported cluster coordinates corresponded to the Montreal Neurological Institute (MNI) coordinate system and were labeled using the AAL labelling atlas in SPM12 (Penny et al. 2011).

#### Region-of-interest extraction

As we had an a priori hypothesis about the mPFC being a region of interest regarding laughter perception, MNI peak coordinates from a prior fMRI study on the perception of conversational laughter versus spontaneous laughter in non-autistic adults (McGettigan et al. 2015) were extracted from three ROIs: (1) left superior medial frontal gyrus (mPFC; *x* = −3, *y* = 54, *z* = 9); (2) left temporal thalamus (*x* = −3, *y* = −6, *z* = 9); and (3) right ACC (*x* = 0, *y* = 30, *z* = 30). To further confirm the mPFC activation, we detected during the processing of conversation laughter and its relationship to the engagement of mentalizing network, an additional analysis was conducted on the meta-analytic mPFC region (*x* = 0, *y* = 50, *z* = 20) reported by Van Overwalle and Baetens (2009). The MarsBaR toolbox (Brett et al. 2002) was used for creating ROIs, building spherical 8-mm radius ROIs around the peak voxels in selected contrasts. Beta values were extracted for analysis.

### Behavioral analysis

The linear mixed model analysis was conducted using R Studio (RStudio 2020) with lme4 package (Bates et al. 2014) to estimate fixed and random coefficients. Model term selection was guided by the Akaike information criterion (AIC; Sakamoto et al. 1986). The car package (Fox et al. 2012) was used to obtain t-statistics, with significance of fixed effects determined using Satterthwaite degrees of freedom. The lmerTest package (Kuznetsova et al. 2017) was used to calculate significance, while the emmeans package (Lenth et al. 2018) was used for Tukey’s honestly significant difference tests and computed the estimated marginal means.

## Results

### Behavioral ratings of words plus laughter

Linear mixed model analysis was conducted to investigate how different types of laughter modulated the perceived funniness of words. Laughter Type (spontaneous vs conversational) and Group (Autism vs NA) were included as fixed effects. We also included participants and word items as two crossed random effects. The models were fitted by restricted maximum likelihood (REML), statistical significance was established via Satterthwaite’s method. There was a significant main effect of Laughter Type ($\beta$ = 0.08, *t*(9496) = 2.94, *P* = 0.003), but not of Group ($\beta$ = 0.22, *t*(9496) = 0.68, *P* = 0.498). This suggests that participants rated words plus spontaneous laughter (*M* = 3.34, *SEM* = 0.164, 95% CI [3.02, 3.67]) as significantly funnier than the words plus conversational laughter (*M* = 3.27, *SEM* = 0.164, 95% CI [2.95, 3.59]). We further included the interaction between Laughter type and Group as a fixed effect to explore whether the laughter modulation effect changes depending on groups. Neither significant interaction effect ($\beta$ = 0.05, *t*(9495) = 0.89, *P* = 0.546) nor significant main effects (Laugh Type: $\beta$ = 0.05, *t*(9495) = 1.48, *P* = 0.138; Group: $\beta$ = 0.20, *t*(9495) = 0.60, *P* = 0.546) were detected from this model. This suggests that autistic and NA participants experienced the laughter modulation effect on the perceived funniness of words similarly (Table 3). Our findings replicate our previous study of the perceived funniness of jokes (Cai et al. 2019).

**Table 3:** Fixed and random effects estimated with the linear mixed model.

Predictors	Estimates	M1		Estimates	M2	
		*CI*	*p*		*CI*	*p*
(Intercept)	3.16	2.71–3.60	<.001	3.17	2.73–3.61	<.001
LaughterType	0.08	0.03–0.13	0.003	0.05	−0.02 – 0.013	0.138
Group	0.22	−0.42– 0.85	0.498	0.20	−0.44– 0.83	0.546
LaughterType × Group				0.005	−0.06– 0.15	0.372
						
Random Effects						
σ^2^	1.64			1.64		
τ_00_	0.15_words_			0.15_words_		
	1.25_participants_			1.25_participants_		
ICC	0.46			0.46		
*N*	48_participants_			48_participants_		
	300_words_			300_words_		
Observations	9502			9502		
AIC	32,309			32,310		
Marginal *R*^2^/conditional *R*^2^	0.004/0.464			0.004/0.464		

*Note. P*-values are given in two tailed.

### Neural responses to implicit processing of words plus laughter versus NV human vocalizations

**Fig. 2:**
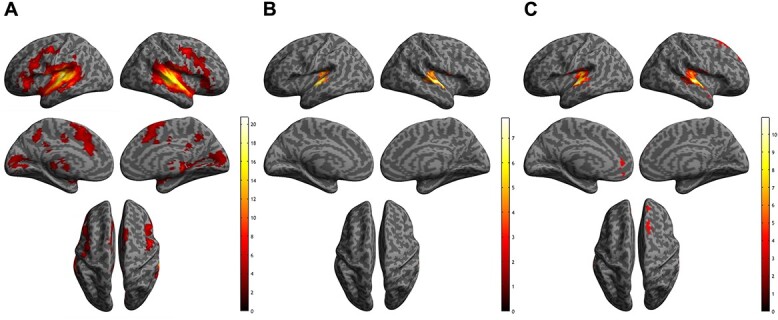
Activations of implicit processing of (A) spontaneous and conversational laughter versus noised-vocoded human vocalization; (B) spontaneous laughter versus noised-vocoded human vocalization; and (C) conversational laughter versus noised-vocoded human vocalization.

We first investigated the neural responses associated with the implicit processing of laughter. This was accomplished by contrasting the activations elicited during the laughter conditions (spontaneous and conversational laughter) with those evoked during the NV human vocalizations condition. Across all participants, whole-brain analyses indicated that hearing words plus both types of laughter versus NV human vocalizations was associated with activation in bilateral Heschl’s gyrus (HG), bilateral STG, bilateral SMA, bilateral posterior (PCC), mid (MCC) and ACC, bilateral precuneus, left middle temporal gyrus, and left superior medial frontal gyrus (mPFC). Additionally, widespread responses were detected in bilateral calcarine, cerebellum, lingual, thalamus, insula, vermis, and inferior frontal gyrus, though the peak coordinates were not located in these regions (See Table 4; Fig. 2A).

**Table 4:** Brain regions showing significant activity in response to implicit processing of laughter versus noised-vocoded human vocalization.

Contrast	No. of voxels	Region	Coordinates				T	Z
			*x*	*y*	*z*			
All Laugh > NV	30,686	Left Heschl’s gyrus	−36	−28	14		20.80	> 8
		Left superior temporal gyrus	−44	−24	8		19.69	> 8
		Left middle temporal gyrus	−66	−26	4		15.63	> 8
		Right Heschl’s gyrus	48	−14	4		18.29	> 8
		Right superior temporal gyrus	60	−12	4		18.86	> 8
	2642	Right supplementary motor area	2	8	64		7.07	5.75
		Right middle cingulate cortex	6	16	38		6.37	5.33
		Left supplementary motor area	−4	14	54		6.33	5.31
		Left middle cingulate cortex	−6	14	40		6.01	5.11
		Left superior medial gyrus	−8	18	40		5.87	5.02
		Left anterior cingulate cortex	−6	26	22		5.64	4.87
			0	24	30		5.14	4.52
	833	Left precuneus	−10	−48	42		5.96	5.08
		Left posterior cingulate cortex	−4	−38	30		4.16	3.80
		Right precuneus	2	−48	38		4.97	4.33
		Right posterior cingulate cortex	4	−38	28		3.92	3.61
	236	Left middle cingulate cortex	−8	−12	40		4.80	4.28
		Right middle cingulate cortex	4	−8	36		3.95	3.63

*Note.* All Laugh = spontaneous and conversational laughter; NV = noise-vocoded human vocalization.

### Neural responses to implicit processing of words plus spontaneous and conversational laughter

Next, we investigated the neural responses associated with implicit processing of different types of laughter. This was accomplished by contrasting the activations elicited during each type of “word plus laughter” condition with those during the NV human vocalization condition (Spont Laugh > NV; Conver Laugh > NV) across all participants. Whole-brain analyses revealed that the spontaneous laughter versus NV condition showed activation in bilateral STG and bilateral Heschl’s gyrus (HG) (see Table 5; Fig. 2B), while the conversational laughter versus NV condition was associated with widespread responses in bilateral superior medial frontal gyri (mPFC), left ACC, left medial orbitofrontal cortex, right superior frontal gyrus (see Table 5; Fig. 2C). No significant group differences (NA > Autism; Autism > NA) were found within these clusters for any of the contrasts in the whole-brain-corrected analysis.

**Table 5:** Brain regions showing significant activity in response to implicit processing of spontaneous laughter and conversational laughter.

Contrast	No. of voxels	Region	Coordinates				T	Z
			*x*	*y*	*z*			
Spont Laugh > NV	1030	Left superior temporal gyrus	−58	−14	2		7.50	5.99
			−58	−22	4		7.15	5.79
		Left Heschl’s gyrus	−42	−18	4		5.33	4.66
	1397	Right superior temporal gyrus	64	−10	4		8.15	6.33
			60	−10	2		8.12	6.32
		Right Heschl’s gyrus	34	−26	16		5.21	4.57
Conver Laugh > NV	1812	Left superior temporal gyrus	−56	−12	4		11.25	7.68
		Left Heschl’s gyrus	−34	−26	16		8.84	6.67
	165	Left anterior cingulate cortex	−12	42	4		4.01	3.68
		Left superior medial gyrus	−4	52	6		3.77	3.49
		Left medial orbitofrontal cortex	−4	46	−14		3.65	3.39
			−8	46	−10		3.49	3.26
	2064	Right superior temporal gyrus	64	−10	4		10.97	7.57
		Right Heschl’s gyrus	36	−26	16		7.47	5.97
		Right superior temporal pole	56	10	−14		4.36	3.95
		Right middle temporal pole	54	10	−24		3.92	3.61
	234	Right superior frontal gyrus	16	26	60		5.06	4.47
			16	12	50		4.72	4.42
	290	Right superior frontal gyrus	18	54	38		4.57	4.11
			16	48	46		4.17	3.81
		Right superior medial gyrus	6	54	34		3.43	3.21

*Note.* Spont Laugh = spontaneous laughter; Conver Laugh = conversational laughter; NV = noise-vocoded human vocalization.

We hypothesized there would be a difference in the neural correlates of the implicit processing of different types of laughter within mPFC and sensorimotor network between groups, as suggested by previous findings. To examine this, we employed a region of interest (ROI) approach. The MNI peak coordinates from a prior fMRI study on the perception of conversational laughter versus spontaneous laughter in non-autistic adults (McGettigan et al. 2015) were used to define the ROIs: left superior medial frontal gyrus (mPFC; *x* = −3, *y* = 54, *z* = 9); left temporal thalamus (*x* = −3, *y* = −6, *z* = 9); and right ACC (*x* = 0, *y* = 30, *z* = 30). For each participant, the mean signal across all voxels was then extracted for the spontaneous laughter versus NV contrast and the conversational laughter versus NV contrast from each of the three ROIs. While significant brain activations in the mPFC were identified in the whole-brain results of implicit processing of conversational laughter across all participants, we adopted an independent approach for hypothesis testing. This ensured that the data used to define the ROIs were not related to the hypothesis testing data, in line with the principles of null hypothesis testing (Kriegeskorte et al. 2009). This unbiased approach also maximized our statistical power to detect group differences within our prior hypotheses.

**Fig. 3:**
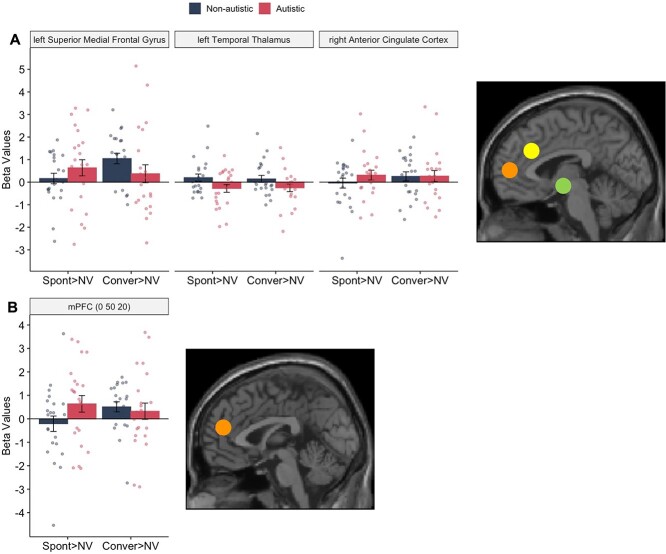
Brain activation and regions in the (A) three ROIs; and (B) metalizing ROI based on meta-analyses between the two groups. *Note.* 3A indicates extracted beta values for ROIs from McGettigan et al. (2015); three brain regions are represented: in orange, the left superior medial frontal gyrus (*x* = −3, *y* = 54, *z* = 9); in green, the left temporal thalamus (*x* = −3, *y* = −6, *z* = 9); and in yellow, the right ACC (*x* = 0, *y* = 30, *z* = 30). 3B indicates extracted beta values for an ROI from Van Overwalle and Baetens (2009); One brain region is represented in orange, the mPFC (*x* = 0, *y* = 50, *z* = 20). Spont = spontaneous laughter; Conver = conversational laughter; NV = noised-vocoded human vocalization. Error bars represent the standard error of the mean.

The 3 (ROIs) × 2 (Contrasts) × 2 (Groups) ANOVA showed a significant three-way interaction (ROIs × Contrasts × Groups), *F*[1.473,64.797] = 5.898, *P* = 0.009, ${\eta}_p^2$ = 0.118, indicating that the interaction among contrasts and ROIs was different between autistic and non-autistic adults. There was also a significant main effect of ROI, *F*[2,88] = 5.499, *P* = 0.006, ${\eta}_p^2$ = 0.111, indicating that the brain activation in these three ROIs was different. Follow-up tests were conducted on each ROI: No significant main effects or interactions were detected in the analysis of the right ACC; a significant main effect of Group was observed in the left temporal thalamus, *F*[1,44] = 4.681, *P* = 0.036, ${\eta}_p^2$ = 0.096, indicating greater activation on both perceptual contrasts in non-autistic (*M* = 0.173, *SEM* = 0.143) than autistic adults (*M* = −.266, *SEM* = 0.143); and a significant two-way interaction (Contrasts × Groups) was detected in the left superior medial gyrus, *F*[1,44] = 7.111, *P* = 0.011, ${\eta}_p^2$ = 0.139. Post hoc analyses revealed significantly greater mentalizing-related BOLD signal change during the implicit processing of conversational laughter compared to spontaneous laughter in the non-autistic group, *F*[1,22] = 9.720, *P* = 0.005, ${\eta}_p^2$ = 0.306 (Spontaneous laughter: *M* = 0.162, *SD* = 1.103; Conversational laughter: *M* = 1.048, *SD* = 1.120). However, there was no significant difference between the two perceptual contrasts in the autistic group, *F*[1,22] = 0.661, *P* = 0.425, ${\eta}_p^2$ = 0.029 (Spontaneous laughter: *M* = 0.637, *SD* = 1.709; Conversational laughter: *M* = 0.374, *SD* = 1.903) (Fig. 3A).

To delve more deeply into whether the mPFC activation identified in aforementioned analyses signifies engagement of the mentalizing network, we conducted a further ROI analysis. We compared the activation of spontaneous laughter and conversational laughter between two groups using a specific mentalizing region of interest, identified through meta-analyses reporting a range of mentalizing tasks which are independent from our current implicit laughter processing task (Van Overwalle 2009; Van Overwalle and Baetens 2009), located at coordinates (mPFC: ×= 0, *y* = 50, *z* = 20). The 2 (Contrasts) × 2 (Groups) ANOVA showed a significant two-way interaction, *F*[1,44] = 4.142, *P* = 0.048, ${\eta}_p^2$ = 0.086, indicating that the activation on two perceptual contrasts differed between groups. Post hoc analyses revealed significantly greater mentalizing-related BOLD signal change during the implicit processing of conversational laughter compared to spontaneous laughter in the non-autistic group, *t*(22) = −2.075, *P* = 0.050, *d* = −.433 (Spontaneous laughter: *M* = −.209, *SD* = 1.571; Conversational laughter: *M* = 0.510, *SD* = 1.026). However, there was no significant difference between the two perceptual contrasts in the autistic group, *t*(22) = 0.847, *P* = 0.407, *d* = 0.176 (Spontaneous laughter: *M* = 0.638, *SD* = 1.689; Conversational laughter: *M* = 0.325, *SD* = 1.666) (Fig. 3B).

Together, these findings suggest that we have replicated previous results on the engagement of the mPFC during the perception of conversational laughter in non-autistic adults (McGettigan et al. 2015). Additionally, by comparing neural differences in mentalizing between autistic and non-autistic groups defined by meta-analytic evidence, our findings further suggest the important role of the mPFC in autistic differences in interpreting *different types* of laughter but not *all types* of laughter.

### Exploratory time-courses of neural responses

**Fig. 4:**
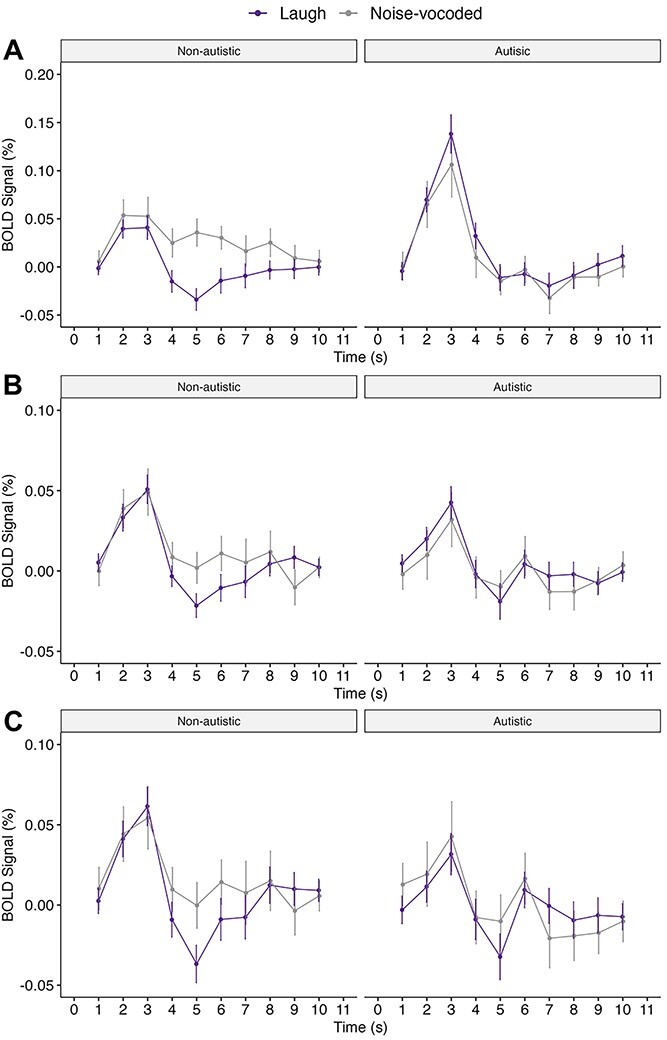
Average time series for all laughter versus NV human vocalization in the (A) SMA, (B) left precuneus, and (C) right precuneus. *Note.* Laugh = spontaneous and conversational laughter; Noised-vocoded = noised-vocoded human vocalization. Error bars represent the standard error of the mean.

We additionally to explore the group differences in the hemodynamic response of brain areas during implicit processing of the words plus laughter conditions and the NV condition, to unpack humor and laughter; we did not have strong predictions about neural differences in the autistic group. Five regions were selected based on a prior interest from the whole-brain results: these correspond to regions associated with hearing sounds and processing speech and emotional vocalizations (bilateral HG and the STG, respectively; Mummery et al. 1999; McGettigan et al. 2015); responses to heard laughter (SMA;McGettigan et al. 2015; O’Nions et al. 2017)) and responses to humor (bilateral precuneus; Li et al. 2018; Brawer and Amir 2021; Chan et al. 2023). The finite impulse response (FIR) event-related time courses were extracted from the above regions, with an FIR length of 30 sec and time bins of 3 s, across all trials of interest. The graphs in Fig. 4 illustrate the greater sensitivity in BOLD signal changes within the SMA and bilateral precuneus areas, but not in HG and STG (Supplementary Fig. S1; HG and STG showed similar profiles), in the non-autistic group during the laughter compared to the NV condition. This contrasted with the autistic group, who demonstrated similar BOLD signal changes in response to both conditions.

To perform a region of interest analysis and compare the two groups’ neural responses within these regions, we adopted a method similar to that used by White et al. (2014). A significant difference was observed only in the SMA area (MNI peak coordinate: *x* = 2, *y* = 8, *z* = 64; 8 mm sphere) between non-autistic (*M* = 0.982, *SD* = 1.175) and autistic (*M* = 2.287, *SD* = 2.099) adults during implicit processing of words plus laughter versus words plus the NV human vocalization, *t*(34.566) = −2.601, *P* = 0.014, *d* = − 0.767.

## Discussion

This is the first study to use fMRI to investigate the neural mechanisms behind the implicit processing of different *types* of laughter in autistic and non-autistic adults. Despite no behavioral differences between the groups, we found that non-autistic adults showed increased activation in the mPFC when listening to words plus conversational than spontaneous laughter, while autistic adults showed no difference in mPFC activity between these two types of laughter. Additionally, autistic adults showed greater activation in SMA than non-autistic adults when listening to words paired with either type of laughter. These findings suggest a critical role for the mPFC and sensorimotor network in the implicit processing of laughter, respectively, in engaging mentalizing to understand socially ambiguous laughter, and in eliciting laughter contagion. Our current findings suggest that autistic people exhibit specific similarities and differences compared to non-autistic people in the implicit processing of laughter. In particular, autistic people experience difficulties in understanding conversational laughter with sophisticated meaning and social ambiguity. It is possible that the challenges autistic people encounter in non-verbal communication seems to not be in processing all social signals, but rather in processing a specific *type* of social signal during communication.

In the whole-brain activation comparing words plus laughter to NV across both autistic and non-autistic groups, we observed neural responses in several regions of the auditory cortex, including bilateral HG and bilateral STG. Additionally, responses were noted in the bilateral PCC, primary visual cortex, precuneus, SMA and mPFC. The recruitment of the dorsolateral temporal lobe fields is consistent with a greater loading on the processing of vocalizations in the words plus laughter conditions (Sander et al. 2005; Fecteau et al. 2007). Our design cannot expand upon this, but it is also possible that there is greater processing in auditory areas than would be seen to spoken words (Mummery et al. 1999; Wise et al. 1999) or laughter alone (McGettigan et al. 2015): the activation in this contrast is extensive in primary and secondary auditory cortex, despite the high-level baseline containing spoken words and NV sounds. It is important to note that the design of our current study differs from previous laughter research (McGettigan et al. 2015; O’Nions et al. 2017): instead of having participants passively listen to laughter stimuli, we used an implicit measure of laughter processing and presented a cover story about people’s responses to a comedian saying funny words, which may have enhanced speech-related processing in auditory fields.

The sensorimotor network, which includes the SMA and ACC as detected in the whole-brain results, has been consistently identified during laughter perception (Warren et al. 2006; McGettigan et al. 2015; O’Nions et al. 2017). This can be attributed to its role as part of a simulation mechanism in facilitating social understanding (McGettigan et al. 2015), contributing to emotional contagion (O’Nions et al. 2017), and its involvement in auditory processing and auditory imagery (Lima et al. 2016). Laughter carries greater socio-emotional weight and is more contagious; therefore increased activity in the sensorimotor network was observed when participants heard words plus laughter compared to NV. Through ROI analyses, autistic adults displayed greater activation in the SMA compared to non-autistic adults, indicating that laughter might be differently contagious for autistic people. This observation seems to align with existing findings highlighting differences in the contagiousness of laughter among autistic people: autistic children are less likely to join in others’ laughter and perceive cartoons with a laughter track as less enjoyable than their peers (Reddy et al. 2002; Helt et al. 2020).

Similar to the involvement of sensorimotor network, the mPFC was consistently observed in the whole-brain results of words plus laughter versus NV, as well as specifically for conversational but not spontaneous laughter. This finding aligns with previous evidence showing greater mPFC activation while listening to laughter with complex social meaning (Szameitat et al. 2010; Sumiya et al. 2017, 2020). In the subsequent ROI results, the non-autistic group showed significantly greater activation in the mPFC during implicit processing of conversational laughter compared to spontaneous laughter. In contrast, no such differential activation was observed in autistic adults. Our finding replicates and extends previous studies on the engagement of the mPFC in processing conversational laughter by including an autistic group (McGettigan et al. 2015; Lavan et al. 2017). Moreover, we went a step further by replicating this difference in neural activation in the mPFC between two groups using a meta-analytic mentalizing ROI (Van Overwalle 2009; Van Overwalle and Baetens 2009). This ROI encompassed a wide range of mentalizing tasks, all designed to tap into a unified cognitive mechanism and independent of our implicit laughter processing task. Our results indicate that the observed mPFC activation reflects the socio-emotional ambiguity of hearing conversational laughter together with funny words. This seems to suggest that non-autistic adults may attempt to resolve the reason for the laughter by engaging in mentalizing, to interpret the meaning and intention behind it.

Further, the lack of mPFC activation in autistic adults during implicit processing of conversational laughter is consistent with Sumiya et al.’s (2020) study, which showed lower mPFC activity in autistic adults compared to non-autistic adults to jokes followed by laughter. Although they did not define their laughter as being spontaneous or conversational, it seems likely that they used the latter. Our results are also consistent with previous findings of atypical function within this mentalizing-related region in autism (Frith and Frith 2006; Gilbert et al. 2009; White et al. 2014). This group difference in autistic adults might reflect their “capacity limits in mentalizing” as proposed by (White et al. 2014), given that we used an implicit measure (adding laughter to modulate the perceived funniness of words) to investigate laughter processing, preventing them from using compensatory strategies. Behaviourally, autistic adults were able to implicitly differentiate between spontaneous and conversational laughter; they rated funny words paired with spontaneous laughter as funnier than the words paired with conversational laughter (see also Cai et al. 2019). There may be multiple potential cues that can be used to implicitly differentiate these two types of laughter. One cue might be related to mentalizing, while another could stem from the inherent acoustic differences between these two types of laughter (Scott et al. 2014; McGettigan et al. 2015; Lavan et al. 2016). It is possible that autistic adults implicitly differentiate laughter based on auditory acoustic properties without engaging in mentalizing. However, our neuroimaging results do not show differences in basic auditory processing. Instead, our data suggest that a reduction in mentalizing may have led to processing laughter without a full understanding of the differences between these two types, and difficulties comprehending the meaning of the laughter in autistic adults. Indeed, subtle differences in the perception of the two types of laughter have been observed during an explicit processing task (explicitly rating the affective properties of two types of laughter); autistic adults rated conversational laughter as more authentic and emotionally arousing, and therefore more similar to spontaneous laughter, than non-autistic adults (Cai et al. in press). Hence, autistic people are able to implicit and explicitly distinguish between these two types of laughter albeit to a lesser extent than non-autistic people, and hence they may struggle to understand the meaning and intentions behind conversational laughter in everyday contexts. This could lead to misunderstandings in social situations, potentially leaving them socially vulnerable (Trundle et al. 2023).

In addition to laughter perception-related areas such as the mPFC and sensorimotor network, we also report significant results associated with the processing of words plus laughter in auditory fields associated with the perception of non-verbal emotional vocalizations and speech, and more notably in the precuneus, posterior bilateral cingulate cortex and primary visual fields. The precuneus has been associated with studies both of mentalizing (Takahashi et al. 2015; Arioli et al. 2021) and of humor (Li et al. 2018; Brawer and Amir 2021; Chan et al. 2023), and its involvement in laughter modulating how funny words seem suggests that it may be an important component in the processes underlying this modulation. The laughter may provoke a further rumination on the meaning of the word, its possible humorous associations, and perhaps the intentions of the person laughing. These reflective processes may relate to the role of the precuneus in metacognitive processes (Ye et al. 2018). The involvement of primary visual cortex in the absence of visual stimulation implies the recruitment of visual imagery to support these processes. Posterior bilateral cingulate cortex has been implicated in studies of autobiographical memory retrieval and emotional salience and is a key node in the default mode network (Leech et al. 2012); its involvement here may indicate both emotional and personal kinds of information being recruited when processing the potentially humorous implications of a word.

The present study has several limitations. Behaviourally, we were unable to collect baseline funniness ratings of words from both autistic and non-autistic adults due to the in-lab testing time restrictions imposed by COVID-19. Given that we measured implicit processing of auditory stimuli, our effects of interests were highly sensitive to background noise, which precluded online testing. Including a baseline measure would enable us to draw more robust conclusions about the similarity of laughter modulation effects between autistic and non-autistic adults. Neurally, though we used laughter to modulate the perceived funniness of funny words in the current study, our design does not distinguish between the processing of laughter and humor. Hence, we further explored group differences by plotting out the time courses of neural responses, to determine if any time-related effects existed, considering the temporal dependency in our stimuli (laughter followed the words). The graphs indicate that, where there are differences between the words plus laughter and NV conditions over time, these occur later in the processing of the stimuli in SMA and precuneus, especially for non-autistic participants. This is consistent with the later integration of the laughter with the verbal material, however, future studies with finer temporal resolution, such as through magnetoencephalography, will better unpack this integration and its overall effects on perceived funniness.

## Conclusion

In sum, this study indicates that autistic people face challenges in processing a specific *type* of social cue during communication, but not in processing all social cues. Our study is the first to use fMRI to probe the neural mechanisms underlying different types of laughter during implicit processing (i.e. how it makes funny words funnier), and, further, to compare the similarities and differences between autistic and non-autistic adults. Despite no behavioral differences between the groups, we found that non-autistic adults exhibited increased mPFC activation when listening to words plus conversational laughter, while autistic adults showed no difference in mPFC activity between these two types of laughter. In addition, autistic adults showed greater activation in SMA than non-autistic adults when listening to words paired with either type of laughter. Our findings suggest a critical role for the mPFC and sensorimotor network in the implicit processing of laughter, respectively in engaging mentalizing to understand socio-emotional meaning of laughter, and in eliciting laughter contagion. Overall, our results highlight the possibility that autistic people may face challenges in understanding the essence of the laughter they frequently encounter in everyday life, especially in processing conversational laughter that carries complex meanings and social ambiguity, potentially leading to social vulnerability. Therefore, we advocate for clearer communication with autistic people.

## Supplementary Material

SI_LaughfMRIAutism_Final_bhae199
