# Supplementary material for: Mapping the differential impact of spontaneous and conversational laughter on brain and mind: an fMRI study in autism
Source: Cereb Cortex. 2024 May 16;34(5):bhae199. doi: 10.1093/cercor/bhae199 (PMC11097909; doi:10.1093/cercor/bhae199)
Supplement: SI_LaughfMRIAutism_Final_bhae199 [file si_laughfmriautism_final_bhae199.docx]

**Supplementary Information**

**Mapping the differential impact of spontaneous and conversational laughter on brain and mind: An fMRI study in autism**

Ceci Qing Cai^1^, Nadine Lavan^2^, Sinead H.Y. Chen^1^, Claire Z.X. Wang^1^, Ozan Cem Ozturk^1^, Roni Man Ying Chiu^3^, Sam J. Gilbert^1^, Sarah J. White^1, +^ and Sophie K. Scott^1, +,*^

^1^ Institute of Cognitive Neuroscience, University College London, WC1N 3AZ.

^2^ Department of Biological and Experimental Psychology, School of Biological and Behavioural Sciences, Queen Mary University of London, E1 4NS.

^3^ Department of Social and Behavioural Sciences, City University of Hong Kong

^+^ SJW and SKS are joint senior authors.

* Corresponding author: Sophie K. Scott

| **Acoustic measure** | **Laughter** | | ***Mean*** | ***SD*** | ***t(df)*** | ***p*** |
| --- | --- | --- | --- | --- | --- | --- |
| Duration | Spont | 2.376 | | .406 | 1.297 (98) | .276 |
|  | Conver | 2.269 | | .361 |  |  |
| Root-mean-square (RMS) | Spont | .317 | | .000 | .908 (98) | .406 |
|  | Conver | .317 | | .000 |  |  |
| Intensity (dB) | Spont | 64.000 | | .000 | .945 (98) | .391 |
|  | Conver | 64.000 | | .000 |  |  |
| Standard deviation of pitch (Hz) | Spont | 90.403 | | 28.697 | .527 (89.860) | .599 |
|  | Conver | 86.785 | | 39.150 |  |  |
| Spectral standard deviation (Hz) | Spont | 866.208 | | 307.739 | -.769 (69.502) | .445 |
|  | Conver | 914.155 | | 144.099 |  |  |
| Fraction of locally unvoiced frames | Spont | 67.083 | | 13.297 | -1.908 (98) | .059 |
|  | Conver | 71.983 | | 12.363 |  |  |
| Pitch | Spont | 389.006 | | 90.403 | 7.801 (98) | **< .001** |
|  | Conver | 279.970 | | 86.785 |  |  |
| Median pitch | Spont | 389.837 | | 77.780 | 8.522 (98) | **< .001** |
|  | Conver | 264.612 | | 68.871 |  |  |
| Spectrum centre of gravity (Hz) | Spont | 1221.347 | | 459.698 | 3.913 (79.123) | **< .001** |
|  | Spont | 926.435 | | 269.498 |  |  |
| Jitter (local) | Conver | 2.952 | | .951 | 5.780 (98) | **< .001** |
|  | Spont | 3.955 | | .776 |  |  |
| Shimmer (local, dB) | Conver | 1.215 | | .284 | -.390 (98) | **< .05** |
|  | Spont | 1.343 | | .254 |  |  |
| Mean harmonics-to-noise ratio (HNR) | Conver | 7.752 | | 2.753 | 4.098 (85.597) | **< .001** |
|  | Spont | 5.836 | | 1.844 |  |  |

**Table S1** Acoustic properties of spontaneous and conversational laughter

*Note.* p values are given in two tailed, signiﬁcant p values are highlighted in bold. Spont = Spontaneous laughter. Conver = Conversational laughter.

**Table S2** Mean of motion parameters of autistic and non-autistic adults within a run

| **Motion parameters** | **Group** | **Mean ± SD** | **t(df)** | **p** |
| --- | --- | --- | --- | --- |
| Translation |  |  |  |  |
| x | Autism | .634 ± .829 | .898(44) | .374 |
|  | NA | .475 ± .196 |  |  |
| y | Autism | .410 ± .236 | 840(32.6) | .407 |
|  | NA | .360 ± .130 |  |  |
| z | Autism | 1.096 ± .706 | .998(44) | .324 |
|  | NA | .925 ± .424 |  |  |
| Rotation |  |  |  |  |
| Pitch | Autism | .871 ± .842 | .275(44) | .784 |
|  | NA | .816 ± .466 |  |  |
| Roll | Autism | .548 ± .373 | .038(44) | .970 |
|  | NA | .552 ± .319 |  |  |
| Yaw | Autism | .422 ± .336 | .233(44) | .817 |
|  | NA | .442 ± .231 |  |  |

*Note.* p values are given in two tailed. Translation in mm. Rotation in radian. NA = non-autistic group.


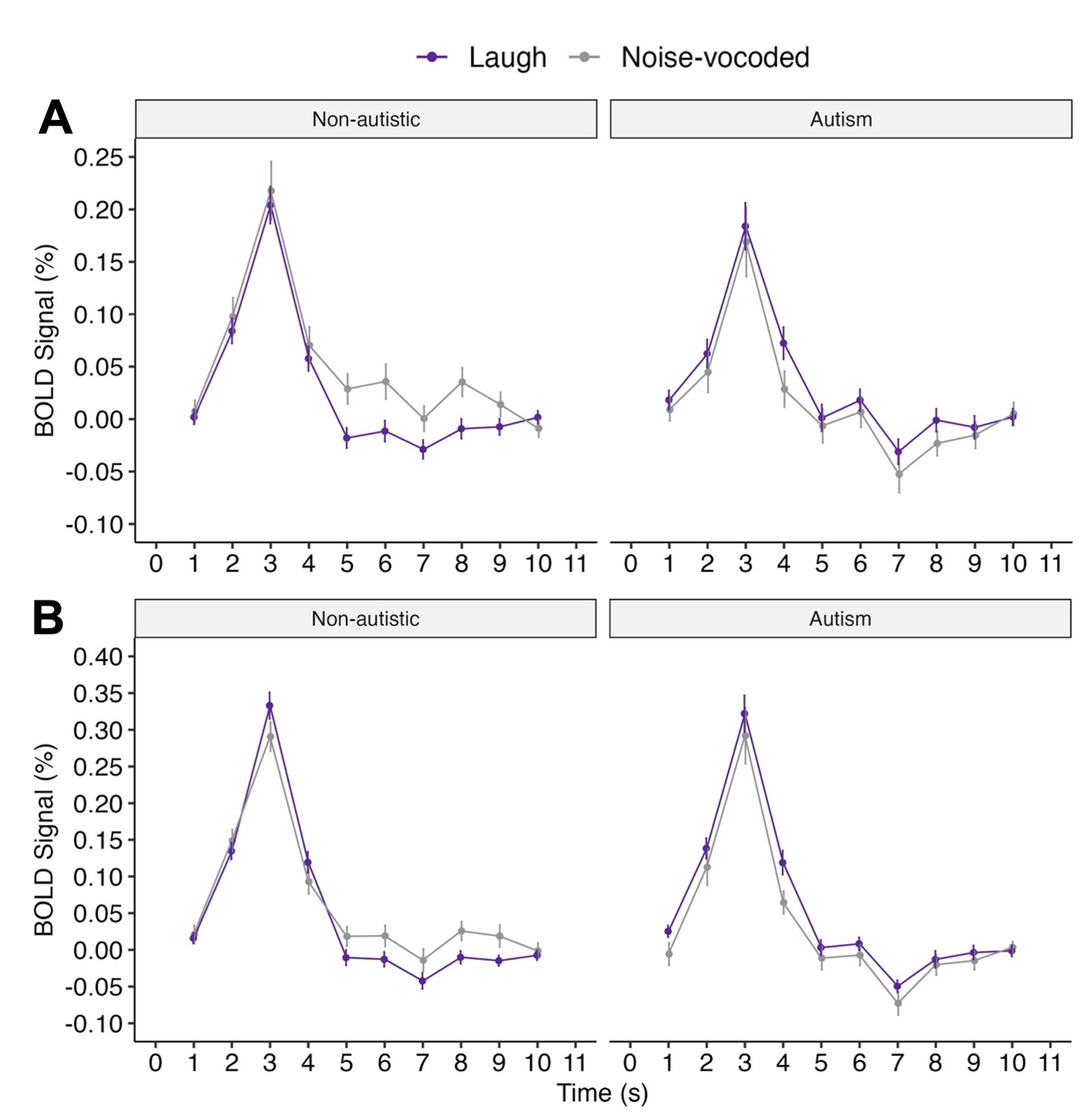


**Figure S1.** Average Time Series for all Laughter vs Noise-vocoded human vocalization in the A) Left STG and B) Right STG.

*Note.* Laugh = spontaneous and conversational laughter. Noised-vocoded = noised-vocoded human vocalization. Error bars represent the standard error of the mean.
